# Evidence on result-based financing in maternal and child health in low- and middle-income countries: a systematic review

**DOI:** 10.1186/s41256-020-00158-z

**Published:** 2020-07-01

**Authors:** Nigel James, Kenny Lawson, Yubraj Acharya

**Affiliations:** 1 Department of Health Policy and Administration, The Pennsylvania State University, University Park, PA 16801 USA; 2 Translational Health Research Institute, Western Sydney University, Sydney, Australia; 3 Hunter Medical Research Institute, Lot 1 Kookaburra Circuit, New Lambton Heights, NSW Australia

**Keywords:** Result-based financing, Maternal and child health care, Low- and middle-income countries, Pay for performance, Institutionalization

## Abstract

**Introduction:**

Result-Based Financing (RBF) is an umbrella term for financial mechanisms that link incentives to outputs or outcomes. International development agencies are promoting RBF as a viable financing approach for the realization of universal health coverage, with numerous pilot trials, particularly in low- and middle-income countries (LMICs). There is limited synthesized evidence on the performance of these mechanisms and the reasons for the lack of RBF institutionalization. This study aims to review the evidence of RBF schemes that have been scaled or institutionalized at a national level, focusing on maternal, newborn, and child health (MNCH) programming in LMICs.

**Methods:**

A systematic literature review was conducted following the Preferred Reporting Items for Systematic Reviews and Meta-Analyses (PRISMA) guidelines. The authors identified and reviewed country-level RBF evaluation reports for the period between January 2000 and June 2019. Data were extracted from both published and gray literature on RBF application in MNCH using a predesigned matrix. The matrix headers included country of application; program setting; coverage and duration; evaluation design and methods; outcome measures; and key findings. A content thematic analysis approach was used to synthesize the evidence and emerging issues.

**Results:**

The review identified 13 reports from 11 countries, predominantly from Sub-Saharan Africa. Performance-based financing was the most common form of RBF initiatives. The majority of evaluation designs were randomized trials. The evaluations focused on outputs, such as coverage and service utilization, rather than outcomes. RBF schemes in all 11 countries expanded their scope, either geographically or accordingly in terms of performance indicators. Furthermore, only three studies conducted a cost-effectiveness analysis, and only two included a discussion on RBF’s sustainability. Only three countries have institutionalized RBF into their national policy. On the basis of the experience of these three countries, the common enabling factors for institutionalization seem to be political will, domestic fund mobilization, and the incorporation of demand-side RBF tools.

**Conclusion:**

RBF evidence is still growing, partial, and inconclusive. This limited evidence may be one of the reasons why many countries are reluctant to institutionalize RBF. Additional research is needed, particularly regarding cost-effectiveness, affordability, and sustainability of RBF programs.

## Introduction

Result-Based Financing (RBF) is an umbrella term covering a number of financing instruments that align incentives to outcomes [1]. Common types of RBF include performance-based financing (PBF), usually referred to as “pay for performance” or P4P; user fees exemptions; voucher programs; and conditional cash transfers (CCTs). These innovative financing instruments utilize the provision of incentives to healthcare providers and/or users to improve health outcomes.

The World Bank is leading the promotion and implementation of RBF projects in maternal, newborn, and child health (MNCH) in low- and middle-income countries (LMICs). The World Bank is also managing the Health Results Innovation Trust Fund (HRITF), a multi-donor trust fund. This fund is supported by the governments of Norway and the United Kingdom [2]. As of September 2016, the HRITF had committed US $385.6 million for 35 RBF programs in 29 countries [3]. Increasingly, other bilateral, multilateral, and philanthropic agencies are channeling some of their funding via RBF [4].

From around the 2000s, the donor community has been funding RBF pilot projects in LMICs, particularly those experiencing a slow progress in the Millennium Development Goals (MDGs) related to maternal and child mortality [5]. RBF is now seen as a strategic health care financing mechanism with the potential to contribute to the achievement of universal health coverage (UHC) [6, 7]. UHC aims to enable all people to access the full spectrum of health care services while protecting them from financial risks associated with seeking these services [8].

Maternal mortality is unacceptably high with the vast majority of the deaths (94%) occurring in low-resource settings [9]. Existing literature suggests that low utilization of MNCH services is due to financial barriers, particularly among the poor [10–12]. Leveraging on effective and efficient health financing models, such as RBF, can potentially increase utilization on the demand side, enhance quality on the supply side, and improve health outcomes. Furthermore, RBF approaches used in MNCH have demonstrated significant increase in coverage and utilization of services [13]. By channeling resources directly to the point of use, RBF mechanisms equip frontline health care providers and managers with the financial capacity and autonomy to institute structural improvements required at the health facilities level, which can eventually improve health outcomes.

Many countries, however, have not institutionalized RBF by integrating such schemes into their national health systems [14, 15]. The reasons for the lack of integration are poorly understood. The aims of this study are to review the RBF schemes that have been scaled from an initial pilot – either geographically or by increasing the scope – and assess the evidence on effectiveness and cost effectiveness, including whether there are documented lessons on potential barriers and enablers to institutionalization. While strong evidence in favor of RBF may not necessarily translate into RBF institutionalization, an emerging body of literature from rigorous large-scale randomized trials has shown that policymakers are indeed receptive to such evidence [16]. Therefore, documenting evidence on the effect of country-level efforts can be an important step in determining the extent to which development agencies should continue to advocate for the institutionalization of RBF.

## Methods

### Study design

The authors conducted a systematic review following the Preferred Reporting Items for Systematic Reviews and Meta-Analyses (PRISMA) guide. The review is registered and published on PROSPERO, an international registry of systematic reviews (ID: CRD42019133119).

### Study setting

This study focused on published and gray literature on country-level RBF evaluation reports. The authors reviewed evaluation reports for various RBF mechanisms being applied in MNCH. Sources were limited to reports from LMICs, which were defined based on the World Bank’s income-based classification [17]. The evaluation reports were predominantly from Sub-Saharan Africa.

### Study period

RBF in MNCH in LMICs is a relatively new concept. Therefore, the authors reviewed RBF studies published between January 2000 and June 2019.

### Search strategy

**Fig. 1:**
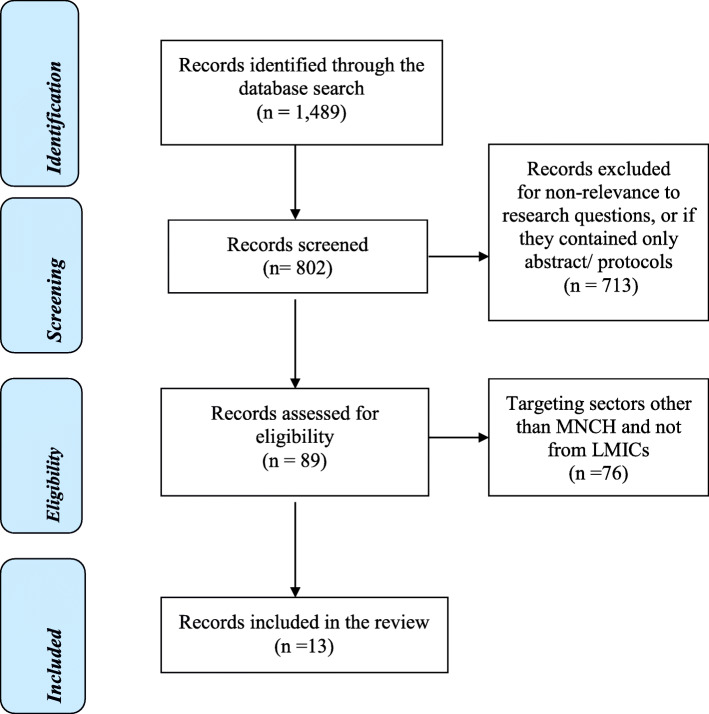
PRISMA flow diagram for the evidence on RBF mechanisms on maternal, neonatal, and child health in low- and middle-income countries

The authors retrieved published country-based RBF evaluation reports using the Web of Science, PubMed, and Google scholar databases following a PRIMSA guideline template (Fig. 1). Relevant records were obtained using the following predetermined search terms: (RBF “OR” Incentives schemes) “AND” (Maternal and Child Health Care “OR” Health Care “OR” Health) “AND” (RBF “OR” Output Based Strategies) “AND” (Impact in MNCH “AND” (RBF programs “OR” RBF projects “OR” Incentives based mechanics “OR” Health Financing “OR” PBF) “AND” (Low- and Middle-Income Countries “OR” Developing Countries).

### Inclusion and exclusion criteria

The inclusion criteria were country-based evaluation reports published between January 2000 and June 2019 for any RBF type in LMICs targeting MNCH and sources being available in English. The exclusion criteria were RBF study evaluation protocols, RBF mechanisms targeting sectors other than MNCH, and studies conducted in non-LMICs settings.

### Data extraction and synthesis

The data from eligible evaluation reports [13] were extracted into a predesigned matrix table. The data included country of application; program setting; program coverage and duration; type of evaluation and methods used; outcome measures; and key findings. The first author (NJ) drafted the consolidated matrix, with the remaining two authors (YA and KL) assessing for consistency and accuracy. In order to evaluate the quality of the reports, the authors adopted the Cochrane Risk of Bias Assessment tool to assess potential selection, performance, and reporting bias. The first author assessed the level of bias (high, low, or unclear), and the co-authors reviewed the assessment. The overall level of bias reported for each study is based on the consensus of all three authors. The framework developed by Shroff and colleagues’ on RBF scale-up was adapted to assess each country’s institutionalization and scale-up progress [15].

## Results

### Sample of studies

The review retrieved 1489 records through the database search (Fig. 1). Of these, 802 were assessed for eligibility. Out of these 802 records, 713 were either not relevant to the research question or did not meet the inclusion criteria, leaving 89 records. Seventy-six of these 89 studies were based on projects that targeted areas other than MNCH or were not conducted in a LMIC, yielding 13 studies for the current review. Of the 13 records, three were based on Rwanda and the remaining 10 country-reports were from Afghanistan, Argentina, Benin, Burundi, Cameroon, Democratic Republic of Congo, Mozambique, Zambia, Zimbabwe and Nigeria (Table 1). Table 2 shows each country’s program scale-up level defined as either generation, adoption, or institutionalization. So far, Rwanda, Cameroon, and Burundi have institutionalized RBF as a national health financing policy.

**Table 1:** Description of RBF evaluation reports including evaluation methods and key findings, by country

Country, Authors, Year	Program Setting	Program coverage	Evaluation timing and program duration	Evaluation method and main outcome measures	Key evaluation findings
Afghanistan Cyrus et al. (2015) [19]	A supply side P4P on selected MNCH indicators in 11 out of 34 provinces. Incentives tied to quantity of care delivered were provided quarterly to healthcare workers	422 health facilities (230– Intervention, − 212–Control)	End-line evaluation Sept 2010- Dec 2012	**Method**: Cluster randomized trial **Outcomes:** Contraceptive prevalence, proportion of deliveries with at least one antenatal care visit, skilled birth attendant, pentavalent 3 vaccination and service utilization	-No substantial effect in any of the five MCH coverage indicators (modern contraception, antenatal care, skilled birth attendance, postnatal care, and childhood vaccination, or in the equity measures -Substantial increases in the quality of history and physical examinations index and the client counselling index, as well as time spent with patients -The inattention to demand-side factors and difficulty in communicating to health workers about the intervention may have undermined the potential effects of the P4P intervention - More attention needs to be given to these factors in the design, management, and implementation of P4P programs
Argentina Gertler et al. (2014) [20]	Supply side P4P national program based on an insurance program that allocated funding to provinces based on enrolment of beneficiaries and adding incentives based on indicators of the use and quality MNCH services	Nationwide	End-line evaluation 2004–2008	**Method**: Cluster randomized trial **Outcomes:** Prenatal care visits, tetanus toxoid vaccine, caesarean section, APGAR score at 5 min	-19% lower chances of low birth weight -74% lower chances in hospital neonatal mortality -Early booking was 34% higher in treatment group with incentives
Benin RBFHealth (2014) [21]	Supply side P4P linked to quantity and quality in 8 out of 34 districts	Four health facilities assigned to intervention arm and one health facility assigned to control	Mid-line evaluation 2010–2011	**Method**: Mixed methods design, consisting of a randomized control trial and qualitative data **Outcomes:** Health worker motivation, ANC services utilization	-Thoroughness of physical examination and history taking in ANC higher in intervention group compared to the control groups -On average, four additional minutes per patient spent on ANC services -No significant effect on productivity or presence of staff in their posts -Greater level of client satisfaction on staff attitude and competence
Burundi Bonfrer et al. (2013) [22]	A supply side PBF program that started off in one province, scaled up to nine before finally being rolled out nationwide	3200 randomly sampled households 75 randomly selected health facilities from intervention and control provinces	End-line evaluation 2006–2008 (Phase 1) 2008–2010 (Phase 2)	**Method:** Repeated cross-sectional survey, analyzed in a difference-in-difference framework **Outcomes**: Institutional delivery, ANC services, vaccination coverages, ITN coverage, child illness episodes, waiting time	-PBF increased the probability of institutional deliveries by 21%, utilization of antenatal care by 7%, and the use of modern family planning methods by 5% -No effect on vaccination rates and user satisfaction -Government committed to allocate 1.4% of its budget to performance-based financing and related health financing strategies each year
Cameroon De Walque et al. (2017) [23]	Payment of health facility bonus linked to volume and quality of services delivered in 14 districts in East, South West and North West regions	14 health districts in the region randomized into four arms as follows: T1- P4P plus autonomy C1- Incentive not attached to performance plus autonomy C2- No incentives at all but autonomy C3- No incentive, no autonomy	End-line evaluation 2012–2015	**Method:** Randomized control trial **Outcomes:** Child and maternal vaccinations, use of modern family planning, antenatal care visits, facility-based deliveries, patient satisfaction	-P4P efficient in bringing payments and funding to provider level, leading to an increased coverage of MNCH and structural measures of quality of care -Decreased OOP payments -No difference in MNCH outcomes between T1 and C1 -No effect observed on skilled deliveries and ANC visits - There was a clear effect of additional financing, irrespective of whether it was linked to incentives
DRC World Bank (2015) [24]	Performance-based payments to health centres and referral centres using a “point system” linked to the volume of targeted services in post conflict Haut- Katanga District- DRC	One out of eight health district zones	End-line evaluation 2009–2013	**Method:** Randomized control trial **Outcomes**: Cost to patients, health workers’ satisfaction, work-related stress and motivation, service utilization, patient satisfaction	-Increased tendency to over report on volumes, but the tendency fell with increased verification -Patient records and data quality better in intervention facilities -Greater transparency and equity in resource allocation among staff -Significant reduction in absenteeism -Increased community-based outreach effort -No change in quality of services in either targeted or non-targeted services -No effect in service utilization -Reduction in job satisfaction -Increased health worker motivation initially, which ultimately reduced intrinsic motivation post intervention
Mozambique Rajkotia et al. (2017) [25]	Phased PBF programs in two provinces Nampula (North) and Gaza (South) targeting 18 MNCH and / HIV-PMTCT services	134 matched facilities health facilities (84 in North, 50 in South)	End-line evaluation 2011- Sept 2013	**Method**: Retrospective data (analyzed using propensity score matching) **Outcomes**: PMTCT, Paediatric HIV indicators vaccination coverages	- The majority of the 18 indicators responded to PBF, with at least half of the indicators showing at least 50% improvement from baseline -Pregnant women indicators (HIV-infected pregnant women initiating ART and family planning consultations for HIV-infected women) were the only adult HIV indicators that responded to PBF -No adverse effects on non-incentivized indicators
Nigeria Kandpal et al., 2019 [26]	PBF and DFF hybrid approach to increase the delivery and utilization of high impact maternal and child health services in three states- Adamawa, Ondo, and Nasarawa	52 Local Government Agencies-LGAs randomised into PBF or DFF and compared with traditional input financing matched states	End-line project evaluation 2012–2016	**Method:** Randomized control trial (for PBF vs DFF comparison) or a quasi-experimental design (for PBF-DFF vs ‘business as usual’ comparison) **Outcomes:** Skilled birth attendance, fully immunized child, modern contraceptive prevalence, pentavalent 3 immunization, institutional delivery, antenatal care visits, equity, CEA	-Significant impact of PBF and DFF on key MCH services as well as quality of care (QOC) (relative to ‘business as usual’). For example, 14 percentage point increase in fully immunized child coverage and 4.5 percentage point increase in use of modern contraceptives -Limited difference in terms of QOC indicators and only a modest difference in terms of MCH services between PBF and DFF -Both interventions found to be cost-effective and likely to be successful due to decentralization of funds, autonomy given to the facilities, improved supervision, and investments in health systems management
Rwanda Basinga et al. (2011) [27]	National supply side PBF program implemented at health facility level.	166 district level facilities randomly selected. (intervention group *n* = 80), (control group *n* = 86)	2006–2010 End line evaluation	**Method:** Randomized control trial **Outcomes:** Prenatal care visits, institutional deliveries, quality of prenatal care, child preventive care visits, immunization	- 23% increases in institutional deliveries in intervention group -56% increase in preventive care visits for 0–23 months age group 132% increase in preventive care visits for 23–59 months age group. - No improvement in the number of women completing four ANC visits or the number of children receiving full immunization - Increased prenatal care quality measured by Rwandan prenatal clinal guidelines - Financial performance incentives can improve quantity and quality of MNCH services and can be in accelerating global development goals
Rwanda (b) Gertler & Vermeersch (2013) [28]	National supply side PBF program implemented at health facility level	166 of Rwanda’s 401 primary care facilities, 80 in treatment districts and 86 in comparison districts.	End-line evaluation 2006–2010	**Method**: Nested randomized control trial **Outcomes**: Health worker productivity, child health outcomes	- Substantial improvements in child health outcomes (weight-for-age and height-for-age z-scores) - Provider incentives led to a 20% increase in productivity - Evidence of complementarity between the incentive and the knowledge (skill) of health care providers
Rwanda (c) Shapira et al. (2017) [29]	Complementary community PBF program that rewarded community health worker cooperatives for the utilization of five targeted maternal and child health ser-vices by their communities		End-line evaluation 2010–2014	**Method**: Randomized control trial **Outcomes:** Nutritional status, use of modern contraceptive methods, ANC and PNC services utilization	-9.6% increased likelihood to attend ANC within 4 months gestational age. -7.2% increased likelihood to attend PNC within 10 days post delivery -Financial rewards to the community health workers did not impact on outcome indicators -No multiplicative effect on outcomes when demand and supply incentives were combined
Zambia Friedman et al. (2016) [30]	Performance based contracting of health centres to deliver a specified package of essential MNCH services.	T1: P4P incentives and medical equipment starter packs C1: Input based grants and medical equipment starter packs C2: nothing was provided.	End-line evaluation 2008–2014	** Method**: Randomized control trial **Outcomes**: Vaccination coverage, job satisfaction, status of infrastructure-drugs and medical equipment, health services coverage-ANC	- T1 and C1 increased in institutional delivery and skilled birth attendances compared to C2. However, more marginal increase was between C1 and C2 -ANC visits were 2 weeks earlier in T1 and C1 compared C2 -Immunization coverage remained the same in T1 but significantly declined in C1 and C2 (P4P – protective factor) -In contrast, PNC was better in C1 compared T1 -Significant structural quality increase in T1 -Health workers in T1 significantly spent more time with their patients during consultations -Patients trusted more T1 services compared to C1 and C2 -Job satisfaction and staff retention were increased in T1 and C1 compared to C2; however, job satisfaction was marginally higher in T1 -No impact on staff motivation in both T1 and C1
Zimbabwe World Bank (2016) [31]	P4P and PBC contracting started in two districts, and in March 2012 was expanded to 16 additional pilot districts, then to 44 country districts	The sample included 16 RBF districts to 16 counterfactual districts (control districts)	Mid-line Evaluation 2011–2014	**Method**: Quasi experimental design, with data analysed in a difference-in-difference framework **Outcomes**: Skilled birth attendance, MNCH service utilization, family planning, vaccination coverages, nutritional status, client satisfaction, OPP, task shifting	-Improvement in skilled providers, in facility deliveries and caesarean sections outcomes; however, this was also the situation generally across Zimbabwe -Program did not have negative effect on non-incentives services -RBF districts had improved autonomy and decentralized decision making -RBF administrative linked tasks aggravated shortage and high workload situation in HF

*Cx* control group, *Tx* treatment group

**Table 2:** RBF scale-up framework

Country	Generation (conducted a pilot project)	Adoption (increased scope of pilot either by adding incentivized indicators or by expanding geographically)	Institutionalization (included RBF as a part of the national health budget planning)
Afghanistan	√	√	
Argentina	√	√	
Benin	√	√	
Burundi	√	√	√
Cameroon	√	√	√
DRC	√	√	
Mozambique	√	√	
Nigeria	√	√	
Rwanda	√	√	√
Zambia	√	√	
Zimbabwe	√	√	

### General features

The program’s implementation duration varied from two to five years. Zimbabwe and Benin reports were mid-line evaluations whereas the rest were end-line evaluations. Most of the studies were conducted as randomized trials; exceptions were those from Benin, Burundi, Mozambique, and Zimbabwe. In the absence of evaluation protocols to check selective reporting, the authors inferred the likelihood of bias based on whether the evaluation team seemed independent from the financing agency. Generally, the level of bias was low to medium (Table 3). The remaining sub-sections provide details on countries’ typical RBF types, evaluation methods, and evidence on cost-effectiveness.

**Table 3:** Risk of bias assessment

Study	Risk Assessment Parameter	Assigned level	Basis of judgment	Assigned overall level
Afghanistan Cyrus et al., 2015 [19]	Random sequence generation (selection bias)	Low risk	Randomized matched pairs	Low Risk
	Allocation concealment (selection bias)	Low risk	Sequence generation and allocation happened simultaneously	
	Blinding of participants and personnel (performance bias)	Low risk	Not feasible due to nature of intervention	
	Blinding of outcome assessment (detection bias)	Low risk	Outcome measures identified before the trial	
	Incomplete outcome data (attrition bias)	Low risk	Cluster level of analysis with all clusters remaining in trial	
	Selective reporting (reporting bias)	Low risk	No evidence of selective outcome reporting, presence of a third-party independent evaluator	
Argentina Gertler et al., 2014 [20]	Random sequence generation (selection bias)	Unclear	“… Over time the membership of the treatment and control group changes.”	Low risk
	Allocation concealment (selection bias)	Low risk	Based on initial phased randomized clinics assignment	
	Blinding of participants and personnel (performance bias)	Low risk	Not feasible due to nature of intervention	
	Blinding of outcome assessment (detection bias)	Low Risk	Difficult to ascertain with multiple outcomes	
	Incomplete outcome data (attrition bias)	Low risk	Cluster level of analysis with all clusters remaining in trial	
	Selective reporting (reporting bias)	Unclear	Mix of independent and non-independent consultants	
Benin RBFHealth, 2014 [21]	Random sequence generation (selection bias)	Low risk	Quantitative component was based on randomized approach	Medium risk
	Allocation concealment (selection bias)	Unclear	Happened at the same time as sequence generation	
	Blinding of participants and personnel (performance bias)	Low risk	Not likely to be a source of bias	
	Blinding of outcome as assessment (detection bias)	Low risk	“… any health staff in the T2 group thought that their bonuses were linked to their performance.”	
	Incomplete outcome data (attrition bias)	Low risk	Not clear	
	Selective reporting (reporting bias)	Unclear	Evaluation team composition not clear	
Burundi Bonfrer et al., 2013 [22]	Random sequence generation (selection bias)	High risk	“...rolled out at the provincial level in a non-randomized way.”	Medium risk
	Allocation concealment (selection bias)	High risk	“...rolled out at the provincial level in a non-randomized way.”	
	Blinding of participants and personnel (performance bias)	Unclear	Not done	
	Blinding of outcome assessment (detection bias)	Unclear	“Facilities receive payments based on the quality of quality of health services provided”	
	Incomplete outcome data (attrition bias)	Unclear	Attrition not discussed	
	Selective reporting (reporting bias)	Low risk	Different independent consultants with different affiliations.	
Cameroon De Walque et al., 2017 [23]	Random sequence generation (selection bias)	High risk	“… was not feasible given that the Government of Cameroon had already decided and announced which districts would be included in the PBF pilot.”	Medium
	Allocation concealment (selection bias)	High risk	Happened at the same time as sequence generation	
	Blinding of participants and personnel (performance bias)	Low risk	Not done	
	Blinding of outcome assessment (detection bias)	Unclear	Difficult to assess given the multiple outcomes	
	Incomplete outcome data (attrition bias)	Low risk	Not likely to be a source of bias	
	Selective reporting (reporting bias)	Unclear	Mix of independent and non-independent consultants.	
DRC World Bank, 2015 [24]	Random sequence generation (selection bias)	Unclear	Not done	High risk
	Allocation concealment (selection bias)	Unclear	Not done	
	Blinding of participants and personnel (performance bias)	Unclear	Not done	
	Blinding of outcome assessment (detection bias)	Unclear	Difficult to assess given the multiple outcomes	
	Incomplete outcome data (attrition bias)	Low risk	Not likely to be source of bias	
	Selective reporting (reporting bias)	Unclear	Part of researchers affiliated to the World Bank	
Mozambique Rajkotia et al., 2017 [25]	Random sequence generation (selection bias)	High risk	“… attempts to control for selection bias using a two-stage approach. First, a matching algorithm was implemented to construct a matched comparison group for all PBF facilities using propensity scores”	Medium risk
	Allocation concealment (selection bias)	Low risk	Not likely to be a source of bias	
	Blinding of participants and personnel (performance bias)	Low risk	Not done	
	Blinding of outcome assessment (detection bias)	High risk	“…. we have no way of determining the extent to which improvements in the intervention group are related to better reporting versus better performance.”	
	Incomplete outcome data (attrition bias)	Unclear	Not likely to be a source of bias	
	Selective reporting (reporting bias)	Low risk	Some researchers declared conflict of interest	
Rwanda (a) Basinga et al., (2011) [27]	Random sequence generation (selection bias)	Low risk	Randomization was done by coin toss	Low risk
	Allocation concealment (selection bias)	Low risk	Happened at the same time as sequence generation	
	Blinding of participants and personnel (performance bias)	Unclear	Not done	
	Blinding of outcome assessment (detection bias)	Unclear	Difficult to ascertain to multiple outcomes	
	Incomplete outcome data (attrition bias)	Low risk	Not likely to be a source of bias	
	Selective reporting (reporting bias)	Low risk	No evidence of reporting bias	
Rwanda (b) Gertler & Vermeersch, 2013 [28]	Random sequence generation (selection bias)	Low risk	“… evaluation employed a stratified cluster randomized designed where districts were first grouped into pairs with common characteristics and then randomly assigned to treatment comparison groups”	Low risk
	Allocation concealment (selection bias)	Low risk	Happened at the same time as sequence generation	
	Blinding of participants and personnel (performance bias)	Unclear	Not done	
	Blinding of outcome assessment (detection bias)	Unclear	Not done	
	Incomplete outcome data (attrition bias)	Low risk	Not follow up cohort design therefore not likely source of bias	
	Selective reporting (reporting bias)	Unclear	Mix of independent and non-independent consultants	
Rwanda (c) Shapira et al., 2017 [29]	Random sequence generation (selection bias)	Low risk	Sectors (sub-districts) in 19 districts were randomly assigned to different study arms	Low risk
	Allocation concealment (selection bias)	Low risk	Not likely to be a source of bias	
	Blinding of participants and personnel (performance bias)	Low risk	Not feasible for the design	
	Blinding of outcome assessment (detection bias)	Low risk	“...to measure outcomes prior to the launch of the program, and to establish internal validity of the study”	
	Incomplete outcome data (attrition bias)	Low risk	“… because the attrition rates were unbalanced between the treatment arms”	
	Selective reporting (reporting bias)	Unclear	Mix of independent and non-independent consultants.	
Zambia Friedman et al., 2016 [30]	Random sequence generation (selection bias)	Unclear	“… selecting districts for the IE was based on district-matched randomization”, however due to budgetary limitations population-based data was only collected in 18 of the 30 study districts, leading to the possible influence of potential unobserved confounders at the district level”	Low risk
	Allocation concealment (selection bias)	Low risk	Happened at the same time as sequence generation	
	Blinding of participants and personnel (performance bias)	Unclear	Not done	
	Blinding of outcome assessment (detection bias)	Unclear	Difficult to ascertain to multiple outcomes	
	Incomplete outcome data (attrition bias)	Low risk	Not likely source of bias	
	Selective reporting (reporting bias)	Low risk	No evidence of bias	
Zimbabwe World Bank, 2016 [31]	Random sequence generation (selection bias)	High risk	“… These 32 districts were purposively sampled from a total of 64 and then pair matched based on observable factors	Medium
	Allocation concealment (selection bias)	High risk	Follows the same risk as random sequence	
	Blinding of participants and personnel (performance bias)	Unclear	Not done	
	Blinding of outcome assessment (detection bias)	Unclear	Difficult considering multiple outcomes	
	Incomplete outcome data (attrition bias)	Unclear	Not reported	
	Selective reporting (reporting bias)	Low risk	No evidence of bias	
Nigeria Kandpal et al., 2019 [26]	Random sequence generation (selection bias)	High risk	﻿ “… design randomly allocated all the 52 LGAs in the experimental states to either the PBF or DFF arms, ﻿however while the PBF versus DFF relies on randomized assignment of LGAs to the two arms, the control comparisons are based on purposively selected states and are quasi-experimental in design”	Medium risk
	Allocation concealment (selection bias)	High risk	Happened at the same time as sequence generation	
	Blinding of participants and personnel (performance bias)	Unclear	Not done	
	Blinding of outcome assessment (detection bias)	Unclear	Difficult to ascertain to multiple outcomes	
	Incomplete outcome data (attrition bias)	Unclear	Not reported	
	Selective reporting (reporting bias)	Low risk	No evidence of bias	

Note: The risk assessment parameters in this study are taken from the Cochrane Risk of Bias Assessment tool. The tool includes additional parameters. Our analysis utilizes six parameters that are commonly used for evaluating randomized trials on public health interventions

### Common RBF approaches

RBF tools can be broadly classified into three categories: supply-side with a demand-side component (focus on provider), demand-side with a supply-side component (focus on provider and consumer), and demand-side with no supply-side component (focus on consumer) [18]. Previous reviews have assessed RBF evidence on one or more of these categories [32]. The country-level studies in the current review predominantly fell under the first category. All 13 studies implemented PBF-type programs that had incentives tied to volume, quality, or both.

Typical program setting involved the contracting of health facilities to offer MNCH services with an incentive tied to quantity, quality, or both. Afghanistan’s PBF intervention targeted health care providers in 230 health facilities, paying bonus payments of up to 10% of existing facility contracts to health facilities based on quantity and quality checklists [19, 33]. Argentina had a similar PBF model, except that payments were made through a national health insurance program that allocated funding to provinces based on enrolment of beneficiaries [20]. Health facility payments in DRC being tied to volume of services provided and not quality was the main difference between the PBF in DRC and those in Argentina, Benin, and Cameroon [20, 21, 23, 24].

The scheme in Rwanda, which was gradually expanded over time, provided both supply- and demand-side incentives. It provided: (i) in-kind incentives (gifts) to women, (ii) performance-based incentives to providers, and (iii) performance-based incentives to community health workers cooperatives for mobilizing mothers to access health services [27, 29]. Nigeria had a unique hybrid of RBF and Decentralized Financing Facility (DFF). In both RBF and DFF approaches, the recipient received direct funding and had autonomy over utilization of those funds. However, in the Nigeria’s DFF, the funds were not linked to quantity or quality of services delivered and the staff did not receive any performance bonuses [26].

### Evaluation methodologies

Evaluation methods differed from country to country. The methods ranged from simple before-and-after comparisons to randomized control trials (RCTs). Of the 13 studies, eight were RCTs, one was a repeated cross section analysis, one was quasi-experimental, one was pre-post comparison, and one was case control. In most studies, randomization was at the level of the facility or higher, and the effects of the interventions were estimated using a difference-in-difference framework (Table 1).

The vast majority of the studies concentrated on output indicators such as antenatal care (ANC) booking rates and percentage of institutional deliveries, with little or no emphasis on quality or impact measures [5, 22]. Eleven countries reported an increase in utilization or coverage because of RBF. For example, a 34% increase in early ANC bookings was recorded in Argentina [20]. Rwanda recorded a 23% increase in institutional deliveries and a 56% and a 132% increase in preventive care visits for children age 0–23 months and 23–59 months, respectively [27].

The effect of RBF on health worker motivation in Zambia and the DRC was mixed. In Zambia, there was no significant improvement in staff motivation, whereas there was a 14% increase in the DRC. However, the effect in the DRC dropped by 25% 4 months after the incentives were removed [24, 30]. RBF in Rwanda and Cameroon had a significant incentive effect in increasing utilization and quality of care for the key MNCH indicators [23, 27]. There was no difference between the PBF and DFF approaches in Nigeria in terms of their effect on the quality of care. However, there were modest differences in the coverage of key services in favor of the PBF approach [26].

### Economic evaluation

The authors analyzed and presented the economic evaluation results following the Consolidated Health Economic Evaluation Reporting Standards (CHEERS) model [34] (Table 4**)**. Three out of the 13 reports (Argentina, Zambia, and Nigeria) included an economic analysis [20, 22, 29]. All three reports provided a Cost Effectiveness Analysis (CEA). The cost-effectiveness estimates were derived from relatively short program implementation periods (2 years and 3 months in Zambia and 4 years in Argentina and Nigeria).

**Table 4:** Economic evaluation results - CHEERS model

Country	Study parameters	Costing	Outcome measurements	Heterogeneity characterization	Estimating tools	Key findings
Zambia Friedman et al., 2016 [30]	Evaluation period-2.25 years Sample size *n* = 338,248 children aged between 0 and 59 months, and 372,073 women of childbearing age. Comparators- C_1_(input financing) C_2_ (no treatment group)	Reported based on programmatic costs (designing, planning implementation and consumables and supplies) Total program costs- US $13.26 million	Quality and coverage of key MNCH indicators-vaccination coverages, family planning, and institutional deliveries	Results not reported for subgroups	Difference in difference approach Lives Saved Tool, QALY	-ICERs were $1642 per QALY gained and $999 per QALY gained, when compared with C1 and C2, respectively, without adjustment for the quality of care -These ratios improve to $1324 per QALY gained and $809 per QALY gained, when compared with C1 and C2, respectively -Program established to be cost effective in terms of lives saved or QALYS gained relative to Zambia’s GDP/ capita in 2013 ($1759) -However, this effectiveness came at a high unit cost
Argentina Gertler et al., 2014 [20]	Evaluation period- 4 years Sample size *n* = 28,042 Unit of analysis -pregnant women and births, Comparators – No treatment group	Reported based on fixed and variable costs (medical equipment, office equipment, vehicles, and administration costs Total program costs-US $106 million	Birth weight and neonatal mortality	Results not reported for subgroups	Difference in difference approach Intention to Treat (ITT) Treatment on Treatment (TOT)	-A DALY saved through PBF in maternal health services were $814 -Program established to be effective in terms of DALYS averted relative to 2005–2008 Argentina GDP/capita of $6075.
Nigeria Kandpal et al., 2019 [26]	Evaluation period- 4 years Unit of analysis -pregnant women and children under 5, Comparators – DFF and C_1_ (no treatment group)	Reported based on PBF implementation costs and costs for designing, implementing, and monitoring Costs were rescaled by population size and calculated as costs per capita. Total program costs-USD $ 132.9 million	Antenatal care, iron supplementation, postnatal care, skill birth attendance, immunization, modern conceptive use, and children slept under insecticide-treated bed nets	Results not reported for subgroups	Difference in difference approach Lives Saved Tool, QALYS	-ICERs of PBF compared to DFF and control were $698 and $796/QALY gained, respectively, without quality of care adjustment -Ratios fell to $458 and $300/QALY gained after adjusting for quality -PBF is cost-effective as compared to the control group regardless of whether life years are adjusted for quality. -Effectiveness of both PBF and DFF is driven by the improvements in the quality of care

When estimating costs, all studies factored in both fixed and variable costs incurred in program design; planning and management; and implementation. The total program costs for programs in Argentina and Zambia were US $106 million and $13.26 million, respectively [20, 30]. The hybrid PBF-DFF program in Nigeria cost US $132.9 million [26].

The reports based on Zambia and Nigeria calculated incremental cost effectiveness ratios (ICERs) comparing PBF to two comparison groups in each case (input financing and no intervention in the case of Zambia and DFF and no intervention in the case of Nigeria) [26, 30]. Depending on the comparison group, ICERs ranged between $809 per QALY gained and $1324 per QALY gained in Zambia (the corresponding range without adjusting for the quality of care was $999 to $1642). Likewise, ICERs ranged between $300 and $458 in Nigeria (between $698 and $796 without adjusting for the quality of care). For Argentina, cost effectiveness was estimated by dividing disability-adjusted life years (DALYs) saved due to RBF by incremental costs of the program. The estimated costs per DALY averted were $814, which was compared to the 2005–2008 per capita GDP of $6075 [20]. All three studies found RBF to be cost effective based on the countries’ annual GDP per capita [20, 26, 30]. This comparison between DALYs or QALYs against the country’s GDP per capita follows the World Health Organization guidelines on the evaluation of public health interventions [35].

## Discussion

Although the development agencies have been encouraging many LMICs to adopt RBF as an important step toward UHC, RBF’s institutionalization remains limited. This study reviewed 13 country-specific RBF evaluation reports from 11 LMICs. In an earlier review similar to this review, Witter et al. [32] concluded that almost all dimensions of RBF impact were understudied for both intended and unintended outcomes. Unlike the earlier review, this review focused on country-level evaluations. While substantially more evidence exists now, the country-level evaluations have primarily focused on outputs rather than outcomes. In the logical framework often used for program evaluation, outputs are the immediate results that are delivered by a program whereas outcomes are the next level of effects resulting from the outputs [36].^1^ Although both measures are useful in understanding the performance of RBF mechanisms, outcomes are more informative since they reveal higher level effects and are more useful for assessing return on investment of the mechanisms.

The improvement observed in structural quality indicators (outputs) at the health facility level is not surprising because RBF mechanisms channel resources to the point of use and foster local autonomy and capacity building.

Only three out of the 13 reviewed reports conducted a cost effectiveness analysis. Given the insufficient evidence on RBF mechanism’s cost effectiveness, the low number of countries to have institutionalized RBF is not surprising. The three studies with a CEA followed the World Health Organization’s GDP per capital threshold method to determine cost effectiveness. Some researchers have argued that this method may not be very useful to decision makers because it might not reflect national budget priorities, values, and country-specific contexts [37]. Nonetheless, evidence from Argentina, Zambia, and Nigeria suggests that RBF yields better returns on investments than traditional input-based financing strategies.

The current RBF implementation arrangements are complex and have high overhead costs, which can jeopardize the affordability and sustainability of RBF mechanisms even if they are deemed to be cost-effective [38]. Witter et al. [32] argue that paying for performance may not always be a good use of resources, even when it is effective, because the potentially small effects are achieved at high costs.

Only two out of the 13 reports in this review included a discussion on sustainability. In Mozambique, on average, it took 18 months of implementation for PBF to show effects, and the impact was generally sustained thereafter [25]. The mobilization of domestic financial resources was central to the sustainability of Burundi’s program [22]. The World Bank, the key proponent of RBF, recommends starting at a low and sustainable level of incentives and gradually increasing them based on a robust financial analysis. The World Bank further recommends that RBF should not be isolated from broader health systems reforms. Instead, it should be viewed as an entry point to tackling system-wide issues [31]. Beyond providing these general directions, the existing literature lacks a meaningful assessment of sustainability of RBF.

Relatedly, most RBF schemes piloted so far are donor funded [39]. Funding agencies view RBF as a good way to reduce the risk of investing funds when there is a possibility of the results not being achieved [39]. Unfortunately, the resulting dependency of the recipient countries on donors compromises the sustainability of RBF programs. If RBF is to make long-lasting impacts in LMICs, an appetite for reform needs to be created within the country. Simultaneously, the capacity to mobilize domestic resources for RBF needs to be built.

On the basis of the experience of the countries that have institutionalized RBF, the common enabling factors for institutionalization seem to be political will, domestic fund mobilization, and incorporation of demand-side RBF tools. For example, in Burundi, the government allocated 1.4% of its budget to PBF each year [22]. Rwanda expanded its PBF program to include a demand-side component that incentivized users [29]. In Cameroon, the government doubled its health sector budget to materialize RBF [23]. Insufficient political will and lack of domestic resources seem to be important challenges to institutionalizing RBF [15], which, of course, may be a reflection of a lack of local ownership and insufficient consideration of resource requirements when RBF is first prescribed to countries.

These findings should be understood in light of a number of caveats. Some of the evaluation studies included in the analysis were not conducted by independent evaluators. Rather, they were conducted by the funding agencies themselves, which raises concerns about the level of bias. The positive effects of RBF are likely weaker than reported in this study. Furthermore, the review only studied sources in English and may have missed relevant studies in other languages. Finally, the interventions analyzed were predominantly on the supply-side, leaving the vast number of financial protection-oriented RBF tools, such as user fees exemptions, voucher schemes, and conditional cash transfers to the users. The latter are important ingredients toward the achievement of UHC [3, 29, 40].

Despite these limitations, the policy implications of these study findings are clear. While political factors may be important in institutionalizing initiatives such as RBF in any country, the evidence on the effectiveness and effects of RBF is so far insufficient. Future research, at a pilot and country level, needs to continuously evaluate RBF schemes, and include qualitative and quantitative research to help define the conditions for successful scale-up, including affordability and sustainability.

## Conclusion

RBF is being promoted as an innovative vehicle toward the achievement of UHC. This review has shown that, while the evidence on the effect of RBF is growing, this evidence is still limited and inconclusive, particularly in areas of cost-effectiveness, sustainability, and system-wide long-term impacts. This limited evidence and low local ownership may be some of the reasons behind countries being reluctant to institutionalize RBF. Additional research is needed, particularly on cost-effectiveness, health system-wide impacts, and sustainability of RBF programs.

## Data Availability

All data generated or analyzed during this study are included in this published article.

## Abbreviations

ANC: Antenatal care
CCT: Conditional cash transfer
CEA: Cost effective analysis
CHW: Community Health Worker
DALY: Disability adjusted life years
DFF: Decentralized financing facility
ICERs: Incremental cost effectiveness ratios
GDP: Gross domestic product
HRITF: Health results innovation trust fund
LGA: Local Government Agency
LMIC: Low and Middle-Income Countries
MNCH: Maternal, Neonatal and Child Health
PBF: Performance based financing
P4P: Pay for Performance
PBC: Performance based contracting
QALY: Quality adjusted life years
QOC: Quality of care
RBF: Result based financing
UHC: Universal health coverage
WHO: World Health Organization

## Footnotes

1 When a program provides financial incentives to health workers tied to institutional births, the change in the proportion of institutional births is the output, whereas the decline in neonatal mortality is the outcome.

## References

[1] Musgrove P. Financial and Other Rewards for Good Performance or Results : A Guided Tour of Concepts and Terms and a Short Glossary. World Bank. 2011;(March):1–4. Available from: http://www.rbfhealth.org/sites/rbf/files/documents/Rewards for Good Performance or Results - Short Glossary.pdf.

[2] Martinez J, Pearson M, Sorenson BH, James B, Sambo C (2012). Evaluation of the Health Results Innovation Trust Fund.

[3] World Bank (2018). World Bank Group support to health services achievements and challenges.

[4] Perakis R, Social Finance. Using Results-Based Funding to Drive Health Equity 2016; Available from: http://www.socialfinance.org.uk/wp-content/uploads/2016/09/SF_ACTION_RBF_Equity_Report_FINAL.pdf.

[5] Grittner AM. Results-based Financing: Evidence from performance-based financing in the health sector. 2013. 1–54 p. Available from: http://www.oecd.org/dac/peer-reviews/Results-based-financing.pdf.

[6] Mills A (2014). Health care systems in low- and middle-income countries. N Engl J Med.

[7] Brenner S, Mazalale J, Wilhelm D, Nesbitt RC, Lohela TJ, Chinkhumba J (2018). Impact of results-based financing on effective obstetric care coverage: evidence from a quasi-experimental study in Malawi. BMC Health Serv Res.

[8] White F (2015). Primary health care and public health: foundations of universal health systems. Med Princ Pract.

[9] WHO. Maternal Mortality. Maternal mortality factsheet. 2018 [cited 2019 Dec 23]. Available from: https://www.who.int/en/news-room/fact-sheets/detail/maternal-mortality.

[10] Borghi J, Ensor T, Somanathan A, Lissner C, Mills A (2006). Mobilising financial resources for maternal health. Lancet..

[11] hIarlaithe MO, Grede N, de Pee S, Bloem M. Economic and social factors are some of the most common barriers preventing women from accessing Maternal and Newborn Child Health (MNCH) and Prevention of Mother-to-Child Transmission (PMTCT) services: A Literature Review. AIDS Behav. 2014;18:516–30. 10.1007/s10461-014-0756-5 24691921

[12] McPake B, Witter S, Ensor T, Fustukian S, Newlands D, Martineau T, et al. Removing financial barriers to access reproductive, maternal and newborn health services: the challenges and policy implications for human resources for health. Hum Resour Health 2013;11(1):1–15. 10.1186/1478-4491-11-46 PMC3849922 24053731

[13] Morgan L. Results-based financing for health performance incentives in global health: Potential and pitfalls FEATURE 1. World Bank. 2016;6. Available from: http://www.rbfhealth.org/sites/rbf/files/RBF_FEATURE_PerfIncentivesGlobalHealth.pdf.

[14] Shroff ZC, Tran N, Meessen B, Bigdeli M, Ghaffar A (2017). Taking results-based financing from scheme to system. Heal Syst Reform.

[15] Shroff ZC, Bigdeli M, Meessen B (2017). From scheme to system (part 2): findings from ten countries on the policy evolution of results-based financing in health systems. Heal Syst Reform.

[16] Hjort J, Moreira D, Rao G, Santini JF (2019). How Research Affects Policy: Experimental Evidence From 2,150 Brazilian Municipalities. NBER Work Pap Ser.

[17] World Bank. World Bank Country and Lending Groups [Internet]. [cited 2019 Nov 21]. Available from: https://datahelpdesk.worldbank.org/knowledgebase/articles/906519-world-bank-country-and-lending-groups.

[18] Gorter A, Ir M, Meessen B. Results-based financing of maternal and newborn health care in low- and lower-middle-income countries. … Matern Newborn Heal … [Internet]. 2013;(February). Available from: https://search.oecd.org/dac/peer-reviews/Evidence-RBF-maternal-health.pdf.

[19] Engineer CY, Dale E, Agarwal A, Agarwal A, Alonge O, Edward A (2016). Effectiveness of a pay-for-performance intervention to improve maternal and child health services in Afghanistan: a cluster-randomized trial. Int J Epidemiol.

[20] Gertler P, Giovagnoli P, Martinez S (2014). Rewarding provider performance to enable a healthy start to life: evidence from Argentina’s Plan Nacer. Policy Res Work Pap World Bank.

[21] RBFHealth. Benin Impact Evaluation. [cited 2019 Dec 23]. Available from: https://www.rbfhealth.org/impact-evaluation/benin-impact-evaluation.

[22] Bonfrer I, Soeters R, Van de Poel E, Basenya O, Longin G, van de Looij F (2014). Introduction of performance-based financing in Burundi was associated with improvements in care and quality. Health Aff.

[23] de Walque D, Robyn PJ, Saidou H, Sorgho G, Steenland M. Looking into the Performance-Based Financing Black Box. Evidence from an Impact Evaluation in the Health Sector in Cameroon. 2017;(August):2–29. 10.1093/heapol/czab002 PMC12147217 33963406

[24] The World Bank. Impact evaluation on Performance Based Financing in Haut-Katanga District, DRC. 2015;1–44. Available from: https://www.rbfhealth.org/sites/rbf/files/Impact Evaluation on PBF in Haut Katanga District - DRC.pdf.

[25] Rajkotia Y, Zang O, Nguimkeu P, Gergen J, Djurovic I, Vaz P (2017). The effect of a performance-based financing program on HIV and maternal/child health services in Mozambique - an impact evaluation. Health Policy Plan.

[26] Kandpal E, Loevinsohn BP, Vermeersch CMJ, Pradhan E, Khanna M, Conlon MK, et al. Impact Evaluation of Nigeria State Health Investment Project. World Bank Gr Open Knowl Repos. 2019.

[27] Basinga P, Gertler PJ, Binagwaho A, Soucat AL, Sturdy J, Vermeersch CM (2011). Effect on maternal and child health services in Rwanda of payment to primary health-care providers for performance: an impact evaluation. Lancet.

[28] Gertler P, Vermeersch C. Using Performance Incentives to Improve Medical Care Productivity and Health Outcomes. NBER Work Pap Ser. 2013:19046 Available from: http://www.nber.org/papers/w19046.pdf.

[29] Shapira G, Kalisa I, Condo J, Humuza J, Mugeni C, Nkunda D, et al. Effects of Performance Incentives for Community Health Worker Cooperatives in Rwanda. 2017;(May):34. Available from: http://documents.worldbank.org/curated/en/573571494939902839/pdf/WPS8059.pdf.

[30] Friedman J, Qamruddin J, Chansa C, Das AK, The World Bank Group (2016). Impact Evaluation of Zambia’s Health Results-Based Financing Pilot Project.

[31] The World Bank (2016). Rewarding provider performance to improve quality and coverage of maternal and child health outcomes.

[32] Witter S, Fretheim A, Kessy FL, Lindahl AK (2012). Paying for performance to improve the delivery of health interventions in low- and middle-income countries. Cochrane Database Syst Rev.

[33] Kandpal E. Completed impact evaluations and emerging lessons from the health results innovation trust fund learning portfolio: World Bank Group - Open Knowledge Repository; 2016.

[34] Husereau D, Drummond M, Petrou S, Carswell C, Moher D, Greenberg D, et al. Consolidated Health Economic Evaluation Reporting Standards ( CHEERS ) statement Stable URL : https://www.jstor.org/stable/42002232 Linked references are available on JSTOR for this article : ( CHEERS ) statement Consolidated Health Economic Evaluation Re. 2019;14(3):367–72. 10.1007/s10198-013-0471-6 23526140

[35] Marseille E, Larson B, Kazi DS, Kahn JG, Rosen S (2015). Thresholds for the cost–effectiveness of interventions: alternative approaches. Bull World Health Organ.

[36] Africa Development Bank. Guidelines for Preparing a Design and Monitoring Framework. Business. 2007. Available from: http://www.adb.org/documents/guidelines/guidelines-preparing-dmf/guidelines-preparing-dmf.pdf.

[37] Bertram MY, Lauer JA, De Joncheere K, Edejer T, Hutubessy R, Kieny M-P, et al. Policy &amp; practice cost–effectiveness thresholds: pros and cons thresholds based on gross domestic product. Bull World Heal Organ. 2016;94(September):925–30 Available from: 10.2471/BLT.15.164418. 10.2471/BLT.15.164418 PMC5153921 27994285

[38] Witter S, Chirwa Y, Chandiwana P, Munyati S, Pepukai M, Bertone MP (2020). Results-based financing as a strategic purchasing intervention: some progress but much further to go in Zimbabwe?. BMC Health Serv Res.

[39] Eldridge M, Tekolste R (2016). Results-Based Financing Approaches Observations for Pay for Success from International Experiences RBF Approaches in Developing Countries.

[40] Parmar D, Banerjee A (2019). How do supply- and demand-side interventions influence equity in healthcare utilisation? Evidence from maternal healthcare in Senegal. Soc Sci Med.

